# Protective Effects of *Dioscorea batatas* Flesh and Peel Extracts against Ethanol-Induced Gastric Ulcer in Mice

**DOI:** 10.3390/nu10111680

**Published:** 2018-11-05

**Authors:** Siyul Byeon, Jisun Oh, Ji Sun Lim, Jeong Soon Lee, Jong-Sang Kim

**Affiliations:** 1 School of Food Science and Biotechnology, Kyungpook National University, Daegu 41566, Korea; sy1_6@naver.com (S.B.); j.oh@knu.ac.kr (J.O.); lzsunny@hanmail.net (J.S.L.); 2 Forest Resources Development Institute of Gyeongsangbuk-do, Andong 36605, Korea; ljs7942@korea.kr

**Keywords:** *Dioscorea batatas* Decne, ethanol-induced damage, gastric ulcer, inflammation, antioxidant

## Abstract

Gastric ulcer is a major digestive disorder and provoked by multifactorial etiologies, including excessive alcohol consumption. In this study, we examined the gastroprotective effect of aqueous and ethanolic extracts of *Dioscorea batatas* Decne (DBD; commonly called Chinese yam) flesh or peel against acidified ethanol-induced acute gastric damage in mice. Our findings demonstrated that oral supplementation of aqueous or ethanolic extracts of DBD flesh or peel before ulcer induction was significantly effective in macroscopically and histologically alleviating ethanol-induced pathological lesions in gastric mucosa, decreasing the plasma levels of inflammatory mediators, such as nitric oxide and interleukin-6, attenuating the gastric expression of cyclooxygenase-2, and increasing the gastric content of prostaglandin E_2_. In particular, pretreatment with the flesh extract prepared in 60% ethanol prominently decreased the expression of biomarkers of oxidative stress, including the plasma levels of 8-hydroxy-2-guanosine and malondialdehyde, and restored heme oxygenase-1 expression and superoxide dismutase activity in the stomach. Overall, these findings suggest that the oral supplementation with DBD extract, especially flesh ethanol extract, prior to excessive alcohol consumption, may exert a protective effect against ethanol-induced gastric mucosal damage in vivo, presumably through the activation of the antioxidant system and suppression of the inflammatory response.

## 1. Introduction

Gastric ulcers are provoked by multifactorial etiologies, such as *Helicobacter pylori* infection, stress, smoking, excessive intake of non-steroidal anti-inflammatory drugs, and excessive alcohol consumption [1,2,3]. 

In particular, ethanol is a damaging agent that causes an acute inflammatory response accompanied by oxidative damage of the cellular components in the gastric wall [4,5]. The damaged region usually exhibits upregulated expression of nuclear factor kappa-light-chain-enhancer of activated B cells (NF-κB), the master regulator of inflammatory responses, and its downstream enzyme cyclooxygenase-2 (COX-2) [6]. In addition, nitric oxide (NO) production is enhanced through the activation of inducible NO synthase (iNOS). The release of proinflammatory cytokines, such as tumor necrosis factor α (TNFα) and interleukin-6 (IL-6), has been associated with the recruitment of macrophages and neutrophils, subsequently leading to gastric inflammation [7].

Extensive research has been carried out to identify the protective potentials of diverse herbs, vegetables, and plants against gastric ulcers [8,9,10]. Numerous plants, or their phytoconstituents, have been found to be effective in relieving the morphological and histological features of gastric ulcerative lesions by increasing the biosynthesis of gastric prostaglandin and downregulating the expression of proinflammatory enzymes. The dietary plant extracts or their components with strong antioxidant activity may exert anti-inflammatory activity via crosstalk between the NF-κB-mediated inflammatory pathway and nuclear factor E2-related factor 2 (Nrf2)-mediated antioxidant defense signaling pathway [11,12,13]. These observations are suggestive of the in vivo protective effects of edible plants against inflammatory response and oxidative stress.

*Dioscorea batatas* Decne (DBD; a synonym for *Dioscorea polystachya* Turcz.), commonly called Chinese yam, has been used as a traditional medicine owing to its several biologically beneficial effects [14,15,16]. Previous studies have demonstrated that Chinese yam exerts strong antioxidative capacity (effectively removing free radicals), and in vitro anti-inflammatory activity (downregulating inflammatory factors) [14,16,17]. In particular, dioscin isolated from *Dioscorea nipponica* was shown to have a protective effect against ethanol-induced liver injury through the reduction of oxidative stress, inflammatory cytokine production, apoptosis, and liver steatosis [18]. 

In the present study, we evaluated the in vivo gastroprotective effects of aqueous and ethanolic extracts of the flesh or peel of Chinese yam and investigated underlying mechanisms in an ethanol-induced gastric ulcer mouse model.

## 2. Materials and Methods

### 2.1. Preparation of Chinese Yam Extracts 

Dry-powdered DBD flesh and peel were obtained from the Forest Resources Development Institute of Gyeongsangbuk-do (Andong, Korea). The flesh or peel powder was extracted in distilled water (DW) or aqueous ethanolic solution (60% or 95% (*v*/*v*) ethanol in DW), as previously described [19]. The extracts were filtered through filter papers (8 μm pore size, Whatman, Little Chalfont, UK), vacuum-evaporated (EYELA N-1000, Tokyo, Japan), and subsequently freeze-dried. The lyophilized extracts were dissolved in ethanol for further examinations.

### 2.2. Development of Ethanol-Induced Gastric Ulcer 

Animal study was approved and conducted by the guidelines of the Institutional Animal Care and Use Committee of the Kyungpook National University (approval number: KNU 2017-151). Six-week-old male Institute of Cancer Research (ICR) mice were purchased from Daehan BioLink (Eumseong, Korea). The experimental animals were maintained under controlled laboratory conditions (temperature of 25 °C, humidity 50 ± 5%, 12 h light/dark cycle). Mice were allowed ad libitum access to drinking water and chow (AIN-76A based cereal feed, Chunhajeil Feed Co., Daejeon, Korea).

After 1 week acclimation, mice weighing 34 ± 2 g were restricted from access to food for 24 h prior to the administration of the water extract (WE) or ethanol extract (EE; extracts in 60% and 95% ethanol were referred to as EE-60 and EE-95, respectively). Each extract was dissolved in vehicle, composed of 5% (*v*/*v*) Tween-80, 10% (*v*/*v*) polyethylene glycol, 10% (*v*/*v*) dimethyl sulfoxide (DMSO), and 10% ethanol in saline, and orally administered at a single dose of either 100 or 200 mg/kg body weight (BW). 

A total of 120 mice were randomized into 15 groups (8 mice per group; Table 1) as follows; group 1 received saline (no ulceration) and vehicle (no treatment), group 2 received acidified ethanol (60% ethanol in saline containing 150 mM HCl; 200 μL/mouse) and the vehicle, group 3 received acidified ethanol and omeprazole (Sigma Aldrich, St. Louis, MO, USA; 20 mg/kg BW), group 4 received acidified ethanol and flesh WE at 100 mg/kg BW, group 5 received acidified ethanol and flesh WE at 200 mg/kg BW, group 6 received acidified ethanol and flesh EE-60 at 100 mg/kg BW, group 7 received acidified ethanol and flesh EE-60 at 200 mg/kg BW, group 8 received acidified ethanol and flesh EE-95 at 100 mg/kg BW, group 9 received acidified ethanol and flesh EE-95 at 200 mg/kg BW, group 10 received acidified ethanol and peel WE at 100 mg/kg BW, group 11 received acidified ethanol and peel WE at 200 mg/kg BW, group 12 received acidified ethanol and peel EE-60 at 100 mg/kg BW, group 13 received acidified ethanol and peel EE-60 at 200 mg/kg BW, group 14 received acidified ethanol and peel EE-95 at 100 mg/kg BW, and group 15 received acidified ethanol and peel EE-95 at 200 mg/kg BW. 

After 3 h resting, mice were subjected to gastric ulceration induction by intragastric instillation of acidified ethanol and were sacrificed after 1 h [20]. The blood and stomach were collected for further histological and biochemical analyses. 

### 2.3. Macroscopic Observation

The removed stomach was cut open along the greater curvature, rinsed in saline, and macroscopically imaged. Gastric lesions were assessed by blinded observers, and scored as follows [21]: 0, no lesions; 1–2, small lesions; 3–4, small ulcer; 5–6, large ulcer; 7, full of ulcers.

### 2.4. Histological Analysis

Collected stomach tissues were fixed in a 10% (*v*/*v*) formalin solution and then paraffin-embedded as previously described [22]. Paraffin blocks were sectioned at 5 μm thickness using a microtome (RM-2125 RT; Leica, Nussloch, Germany). Tissue sections were stained with hematoxylin and eosin (H&E) dyes. Tissue damage was observed under a microscope (Eclipse 80i; Nikon, Tokyo, Japan). 

### 2.5. Measurement of 8-Hydroxy-2’-Deoxyguanosine (8-OHdG) Level

The plasma 8-OHdG concentration was determined using an enzyme-linked immunosorbent assay (ELISA) kit (Enzo Life Sciences, Inc., Farmingdale, NY, USA) as previously described [22]. Briefly, 50 μL of plasma samples, or a series of standard dilutions, were dispensed into each well of an immunoassay plate. The samples were allowed to react with a primary antibody against mouse 8-OHdG and, subsequently, with a horse radish peroxidase (HRP)-conjugated anti-mouse IgG. Antibody reactivity was visualized using 3, 3’, 5, 5’-tetramethylbenzidine (TMB) substrate. The absorbance was measured at 450 nm using a microplate reader (Sunrise™, Tecan Group Ltd., Männedorf, Switzerland).

### 2.6. Determination of Malondialdehyde (MDA) Level

The stomach tissues were homogenized in lysis buffer (0.1 M phosphate buffer, pH 7.4) and centrifuged at 10,000× *g* for 30 min at 4 °C. The supernatant was used to quantify the content of thiobarbituric acid reactive substances (TBARS) using an assay kit (Cat # ALX-850-287; Enzo Life Sciences, Inc., Farmingdale, NY, USA) according to the manufacturer’s instructions. After reaction completion, the absorbance was detected at 532 nm.

### 2.7. Determination of Gastric Prostaglandin E_2_ (PGE_2_) Level

The supernatant of stomach tissue homogenate was subjected to measurement of PGE_2_ level using an ELISA kit (Cat # 514010; Cayman chemical, Ann Arbor, MI, USA). The stomach tissues were homogenized in lysis buffer (0.1 M phosphate, pH 7.4, containing 1 mM EDTA, and 10 μM indomethacin) followed by centrifugation at 10,000× *g* for 30 min at 4 °C. The level of PGE_2_ in the homogenate was quantified by interpolating the absorbance values at 420 nm to a standard curve.

### 2.8. Determination of Catalase (CAT) and Superoxide Dismutase (SOD) Activities

The supernatant of stomach tissue homogenate was subjected to analysis using commercially available kits; CAT ELISA kit (Roche, Basel, Switzerland) and SOD assay kit (Dojindo Molecular Technologies, Inc., Rockville, MD, USA), as previously described [23,24]. 

### 2.9. Determination of Plasma Nitric Oxide (NO) and Cytokine Levels

The plasma NO concentration was determined by measuring the nitrite content using the Griess reagent system (Promega, Madison, WI, USA). The levels of cytokines were quantified using ELISA kits for IL-6 and TNFα (all from BD Biosciences, San Diego, CA, USA), as previously described [22]. 

### 2.10. Western Blot Analysis

Nuclear and cytoplasmic proteins in the tissue homogenate were fractionated using NE-PER^TM^ Nuclear and Cytoplasmic Protein Extraction Kit (Thermo Fisher Scientific., Rockford, IL, USA), as previously described [22]. After quantification and denaturation, the proteins were electrophoretically separated on a sodium dodecyl sulfate polyacrylamide gels, and the separated bands were transferred onto polyvinylidene fluoride membranes (Merck Millipore Corp., Billerica, MA, USA). The primary antibodies used in this study were directed to COX-2 (Cell Signaling Technology, Danvers, MA, USA), heme oxygenase-1 (HO-1; Abcam, Cambridge, UK), or β-actin (Santa Cruz Biotechnology, Dallas, TX, USA). Appropriate secondary antibodies, HRP-conjugated, were used for each primary antibody. Protein bands were developed, digitalized, and densitometrically analyzed using Image Studio Lite version 5.2 (LI-COR Biotechnology, Lincoln, NE, USA).

## 3. Statistical Analysis

All the statistical analyses were performed using the SPSS software version 23.0 (SPSS Inc., Chicago, IL, USA). Comparisons were conducted via one-way analysis of variance (ANOVA) followed by Duncan’s multiple range test. The *p*-values less than 0.05 were considered significant. Significant differences were indicated using different alphabetical letters.

## 4. Results

### 4.1. DBD Flesh and Peel Extracts Alleviated Acidified Ethanol-Induced Gastric Mucosal Damage

**Figure 1:**
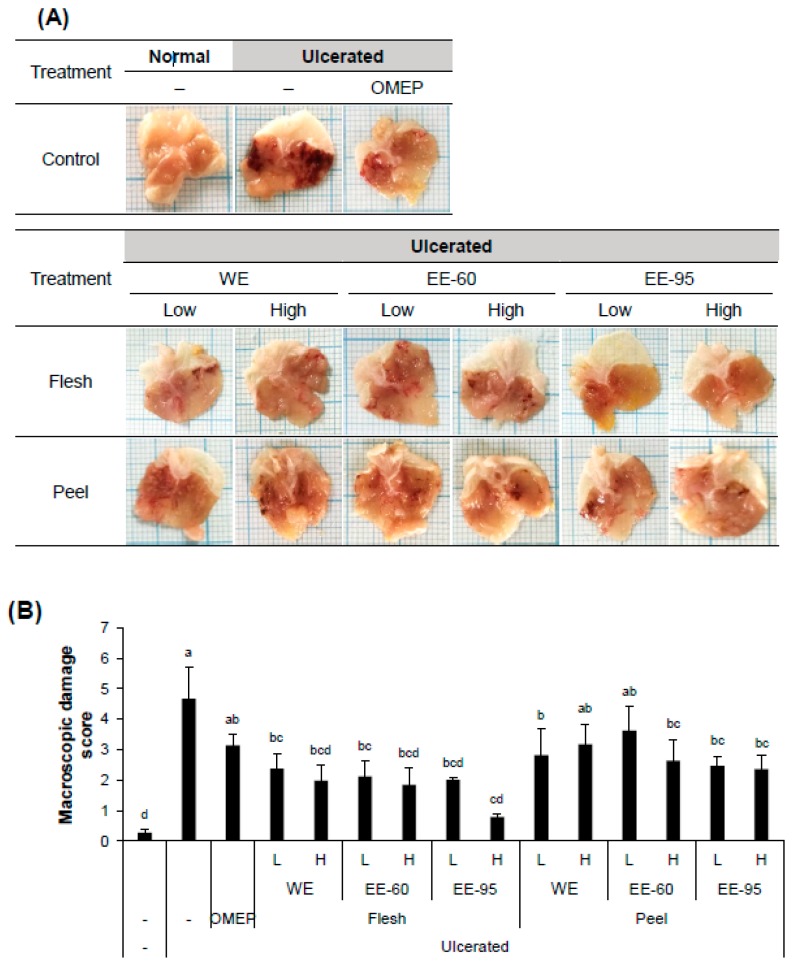
*Dioscorea batatas* Decne (DBD) flesh and peel extracts improved the macroscopic morphology of gastric damage induced by HCl/ethanol in mice. ICR mice were randomly assigned to 15 different groups (8 mice per group). The water or ethanol extracts of flesh or peel were orally and singly administered, at a designated dose, 3 h prior to an intragastric infusion of ethanol, to induce acute gastric ulceration. After 1 h, mice were sacrificed. (**A**) Representative photos of the dissected stomach. (**B**) Score for macroscopic gastric damage. The data obtained from individual animal samples per group were averaged (*n* = 8); values represent mean ± standard deviation (SD). OMEP, omeprazole used as a positive control. WE, water extract; EE-60, ethanol extract in 60% ethanol; EE-95, ethanol extract in 95% ethanol. Bars not sharing common letter represent statistically significant difference from each other (*p* < 0.05).

**Figure 2:**
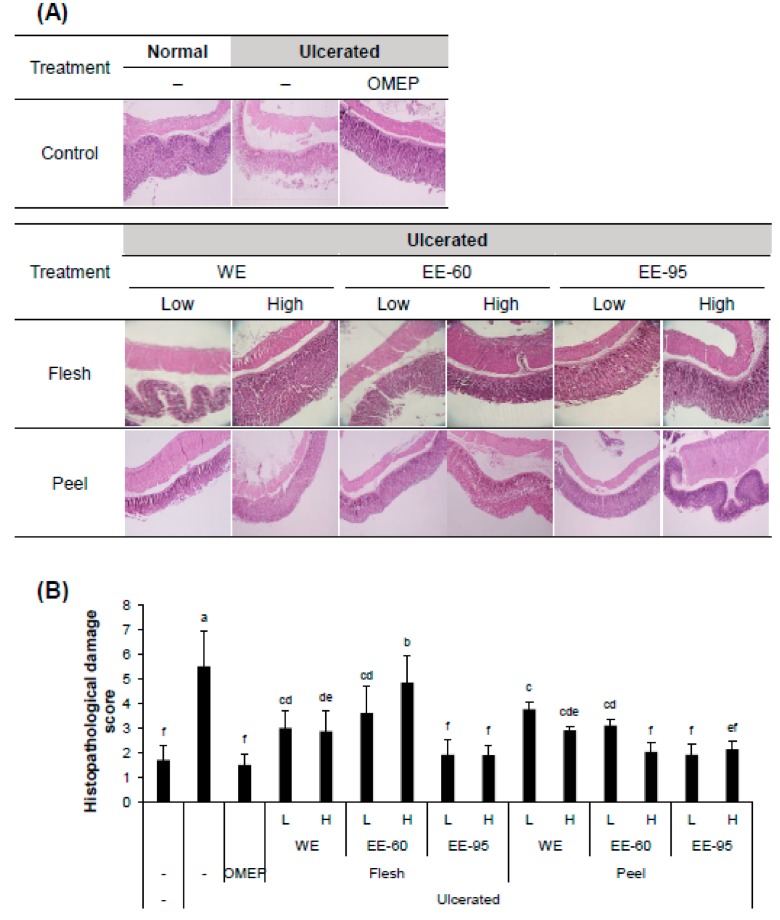
DBD flesh and peel extracts alleviated HCl/ethanol-induced gastric epithelial damage. The dissected stomach tissue was fixed in formalin solution, embedded in paraffin, sectioned using a microtome, and stained with hematoxylin and eosin (H&E) for histological analysis. (**A**) Representative photomicrographs of gastric mucosal surface (magnification, 40×). (**B**) Score for gastric mucosal damage. Values represent mean ± SD (*n* = 8). OMEP, omeprazole used as a positive control. WE, water extract; EE-60, ethanol extract in 60% ethanol; EE-95, ethanol extract in 95% ethanol. Bars not sharing common letter represent statistically significant difference from each other (*p* < 0.05).

To examine the gastroprotective effect of DBD flesh or peel extract (either water or ethanol) in vivo, adult male ICR mice were pretreated with the extracts 3 h prior to the induction of acute gastric ulcer by the intragastric instillation of acidified ethanol. After 1 h, mice were sacrificed followed by observations on macroscopic and histological damage of the gastric mucosa (Figure 1 and Figure 2).

Acidified ethanol, as expected, induced a large area of hemorrhagic ulcerative gastric lesions (Figure 1A). The severity of the damage was significantly decreased in mice pretreated with both water and ethanol extracts of DBD flesh as well as in mice pretreated with ethanol extract of DBD peel (Figure 1B). Furthermore, ethanol administration caused significant alteration in the gastric epithelium, reflecting hemorrhagic necrosis and collapse of the gastric mucosa with epithelial cell loss (Figure 2A). The pretreatment with DBD flesh and peel extracts significantly alleviated ethanol-induced epithelial damage and contributed to preserving the structure of gastric wall, as evidenced by the low histopathology score (Figure 2B). These macroscopic and histological examinations indicate that both water and ethanol extracts of DBD flesh or peel ameliorated ethanol-induced gastric mucosal damage.

### 4.2. DBD Flesh and Peel Extracts Reduced Acidified Ethanol-Induced Oxidative Stress

**Figure 3:**
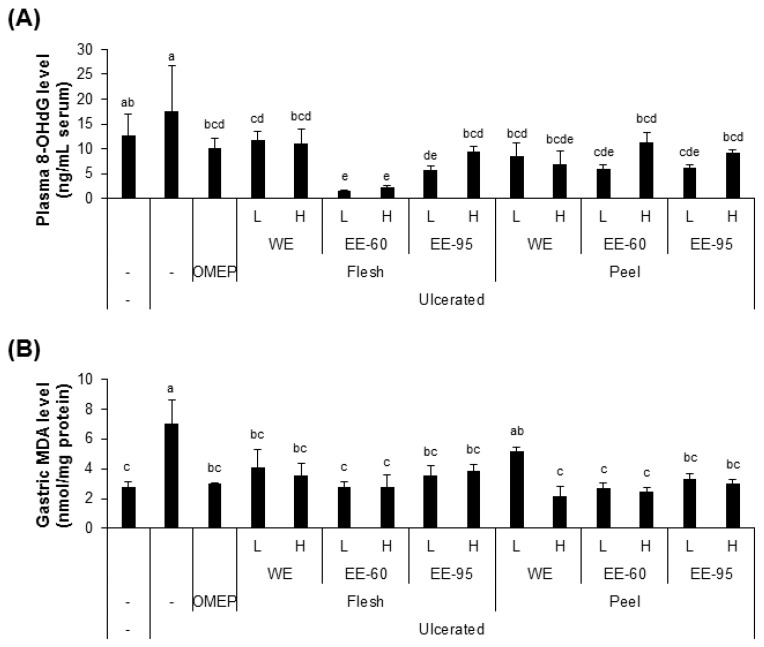
DBD flesh and peel extracts reduced HCl/ethanol-induced oxidative stress. The levels of 8-OHdG in the serum (**A**) and malondialdehyde (MDA) in the stomach homogenate (**B**) were elevated by ulcer induction and lowered by pretreatment with the extracts. Values represent mean ± SD (*n* = 8). Bars not sharing common letter represent statistically significant difference from each other (*p* < 0.05).

Since the gastric injury caused by ethanol is typically accompanied with oxidative stress in the gastric tissues [25], we examined the levels of biomarkers of oxidative stress, including 8-OHdG (a marker of oxidative DNA damage) in the serum and MDA (an end-product of lipid peroxidation) in the gastric homogenates (Figure 3). The plasma 8-OHdG and gastric MDA concentrations were significantly elevated by ethanol administration (Figure 3A,B), while pretreatment with both water and ethanol extracts of DBD flesh or peel suppressed the elevation of these biomarkers. These findings suggest that water and ethanol extracts of DBD flesh or peel may attenuate the oxidative stress induced by acidified ethanol in the mouse model of acute gastric ulcer.

**Figure 4:**
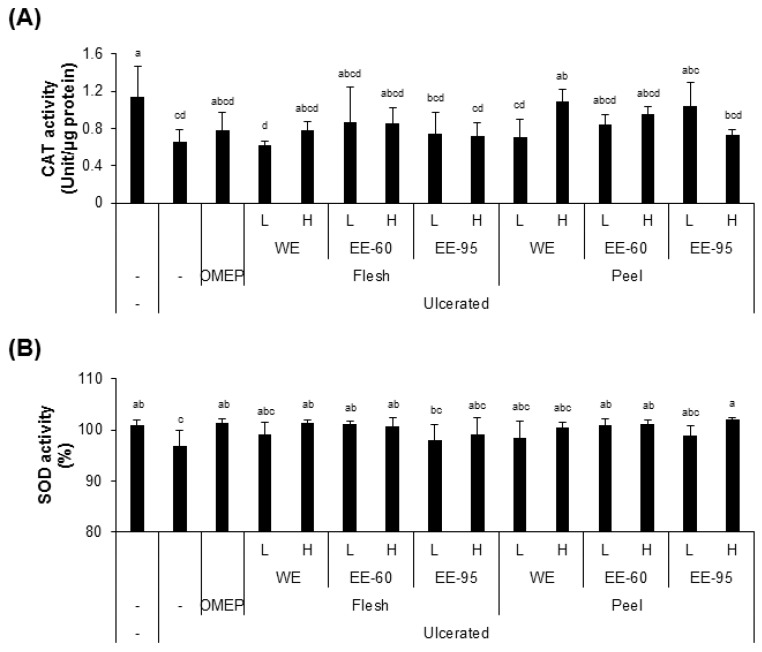
Effect of pretreatment with DBD flesh and peel extracts on catalase (CAT) and superoxide dismutase (SOD) activities in stomach homogenate. (**A**,**B**) HCl/ethanol treatment resulted in significant reduction in the activities CAT and SOD. Pretreatment with WE or EE restored the enzyme activities, but not significantly at the 0.05 level. Values represent mean ± SD (*n* = 8). Bars not sharing common letter represent statistically significant difference from each other (*p* < 0.05).

However, these extracts somewhat, but insignificantly, recovered the activities of antioxidant enzymes, such as CAT and SOD in the serum, that were decreased by ulcer induction by ethanol instillation (Figure 4A,B). Notably, pretreatment with the extract in 60% ethanol (EE-60) of DBD flesh resulted in a remarkable reduction in 8-OHdG level, and a complete recovery of SOD activity, despite ulcer induction.

### 4.3. DBD Flesh and Peel Extracts Decreased Acidified Ethanol-Enhanced Production of Inflammatory Factors

**Figure 5:**
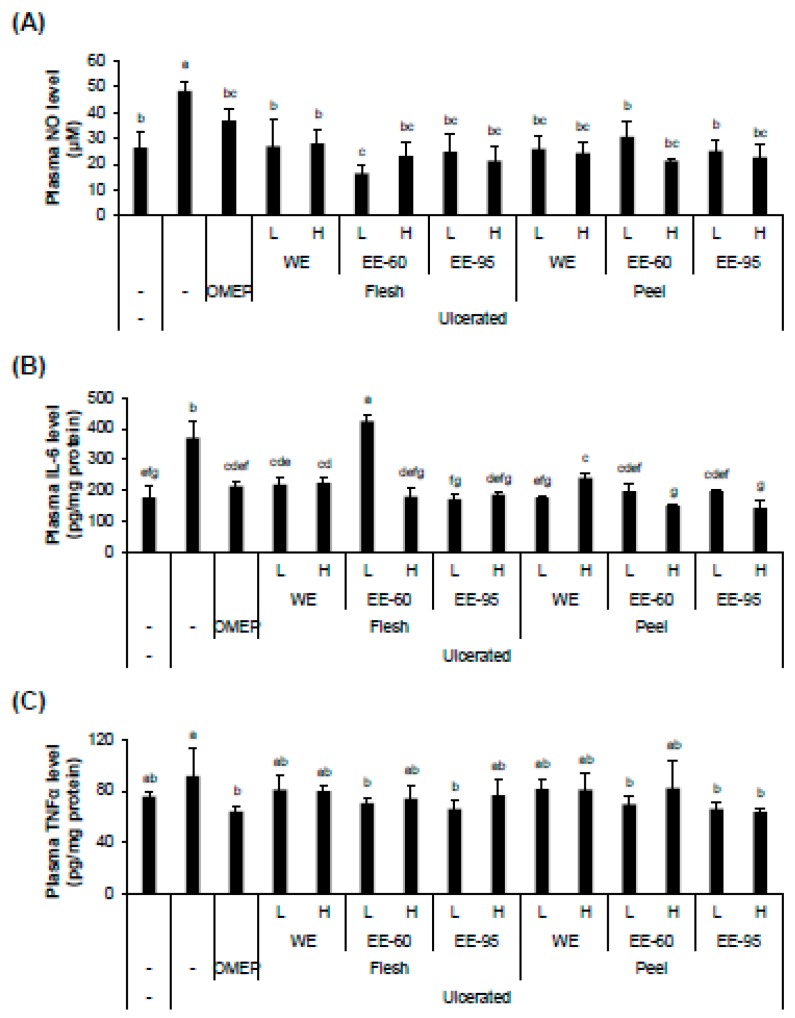
DBD flesh and peel extracts decreased HCl/ethanol-induced production of inflammatory factors. The serum samples were collected after sacrificing HCl/ethanol-treated mice. The plasma levels of NO and proinflammatory cytokines (IL-6 and TNFα) were measured by ELISAs. The increased levels of plasma NO (**A**) and IL-6 (**B**) by gastric ulcer induction were significantly decreased by pretreatment with both WE and EE. However, TNFα levels (**C**) in the experimental groups were considerably reduced by pretreatment with flesh or peel EE, but not with WE. Values represent mean ± SD (*n* = 8). Bars not sharing common letter represent statistically significant difference from each other (*p* < 0.05).

Although recent studies have highlighted the anti-inflammatory effect of *Dioscorea* spp., the mechanism underlying the protective effect against ethanol-induced damage is incompletely understood [26,27,28]. We examined the anti-inflammatory activity of DBD extracts in acute gastric ulcer in mice by evaluating the plasma levels of NO, IL-6, and TNFα (Figure 5). Both water and ethanol extracts of DBD flesh or peel decreased the concentrations of inflammatory factors, including NO and IL-6, in the serum (Figure 5A,B). In addition, the plasma TNFα level, enhanced by ulceration, was significantly decreased by pretreatment with ethanol extracts of flesh or peel, but not with their water extracts (Figure 5C). 

### 4.4. DBD Flesh and Peel Extracts Influenced the Expressions of an Inflammatory Marker COX-2 and an Antioxidant Enzyme HO-1 in Gastric Mucosa

**Figure 6:**
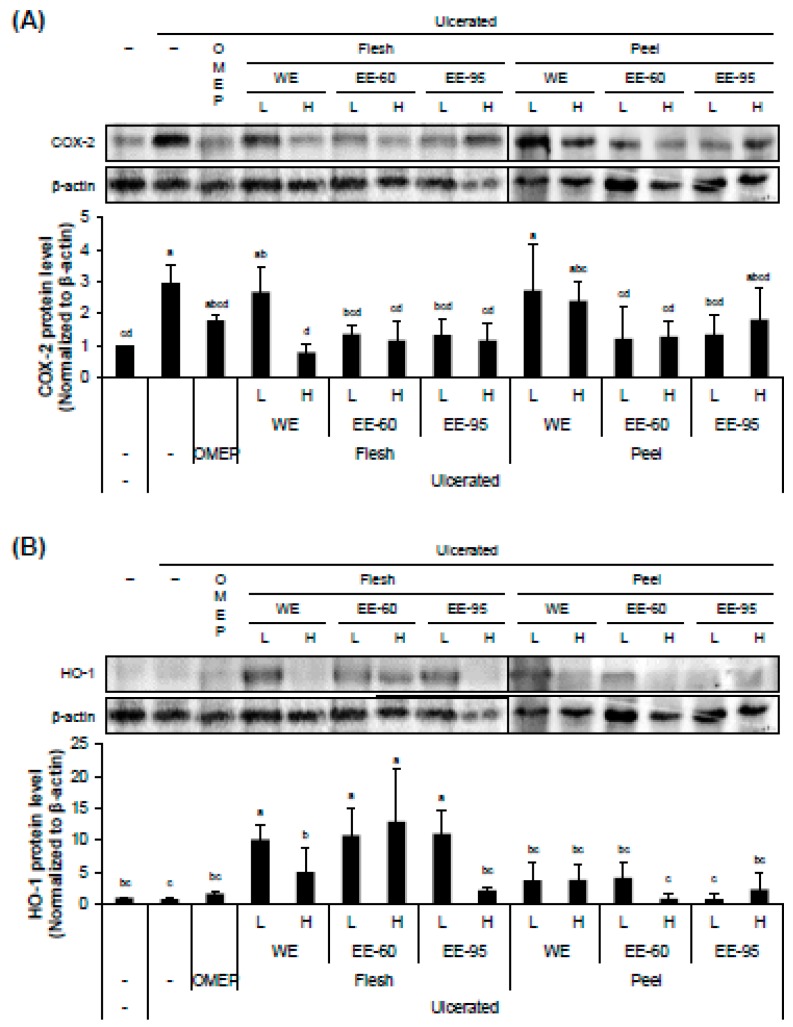
DBD flesh and peel extracts influenced the expressions of the inflammatory marker COX-2 and the antioxidant enzyme HO-1 in gastric mucosa. Stomach homogenates were used for the analysis of protein expression of COX-2 (**A**) and HO-1 (**B**). Both WE and EE of flesh and EE of peel reduced the increased expression of COX-2 in HCl/ethanol-treated stomach. However, HO-1 protein level increased only by pretreatment with flesh extracts; in particular, EE was more potent than WE. Peel extracts showed no significant influence on HO-1 expression. Values represent mean ± SD (*n* = 8). Bars not sharing common letter represent statistically significant difference from each other (*p* < 0.05).

The expression level of COX-2 in the stomach tissue was obviously increased by ethanol administration and significantly reduced by pretreatment with ethanol extracts of DBD fresh or peel (Figure 6A). On the other hand, the expression level of an antioxidant enzyme HO-1 was little influenced by intragastric ethanol instillation, but remarkably increased by pretreatment with ethanol extracts of DBD flesh (Figure 6B). Interestingly, the ethanol extract of DBD peel which was effective in lowering the acidified ethanol-enhanced COX-2 expression, had no significant effect on the expression of HO-1. These observations suggest that the flesh and peel extracts of DBD exert their anti-inflammatory effects through distinct pathways. 

### 4.5. DBD Flesh and Peel Extracts Increased Acidified Ethanol-Lowered Level of PGE_2_ in Stomach Homogenate

**Figure 7:**
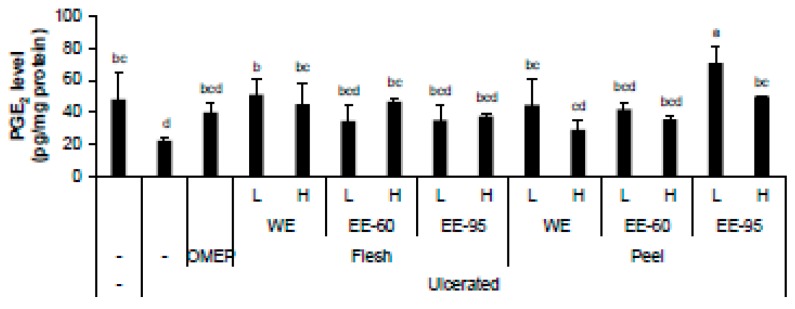
DBD flesh and peel extracts increased the HCl/ethanol-lowered level of PGE_2_ level in stomach homogenate. The gastric PGE_2_ level was prominently decreased by ulcer induction, but the oral administration of the extracts resulted in changes in PGE_2_ level. In particular, pretreatment with flesh WE and peel EE-95 significantly restored PGE_2_ level. Values represent mean ± SD (*n* = 8). Bars not sharing common letter represent statistically significant difference from each other (*p* < 0.05).

PGE_2_ is known to be endogenously produced in the gastrointestinal mucosa and play a critical role in the healing of gastric ulcer, which is likely to be mediated by EP4 receptor [29]. The level of PGE_2_ in stomach homogenate was reduced when ulcerated by acidified ethanol; however, pretreatment with the water extract of flesh or the ethanol extract of peel restored the levels of PGE_2_ (Figure 7).

## 5. Discussion

*Dioscorea* species, belonging to the family Dioscoreaceae, are mostly grown for their starchy tubers or medicinal properties [26,28,30]. DBD is known to have biologically beneficial effects including antioxidative, antidiabetic, anti-atherosclerotic, and anti-inflammatory activities [14,16,17,27,31,32]. In particular, the ethanol extract of DBD peel is known to inhibit the production of NO and PGE_2_ in lipopolysaccharide-induced Raw 264.7 cells [16]. Supplementation of DBD flesh powder in diet enhanced antioxidant enzyme activities and reduced colonic mucosal inflammatory mediator gene expression in azoxymethane-treated F344 rats [27]. Despite the evidences demonstrating the diverse health-promoting benefits of DBD, little is known about the positive effect of its flesh and peel on gastric injury. 

In order to examine the gastroprotective effect of dietary intake of DBD flesh or peel extracts, acute gastric ulcer was induced in adult male ICR mice by intragastric infusion of acidified ethanol with or without DBD pretreatment. Acidified ethanol administration causes acute hemorrhagic gastric damage attributed to increased neutrophil infiltration, which is the sign of inflammatory reaction [33,34,35]. Omeprazole, an anti-ulcer agent, was used as a positive control, given its popular prescription for the treatment of gastric ulcer [36,37].

The present study demonstrated that oral supplementation with both water and ethanol extracts of DBD flesh and peel protected the mouse gastric mucosa against acidified ethanol-induced injury by decreasing the levels of inflammatory factors, including NO and IL-6 in the serum and COX-2 expression in the gastric tissue, and by increasing the production of gastric PGE_2_. In particular, the flesh extract prepared in 60% ethanol significantly restored the gastric HO-1 protein expression and SOD enzyme activity, and reduced oxidative stress biomarkers (plasma 8-OHdG and gastric MDA levels), indicative of the increase in the antioxidative capacity. These findings suggest that the ethanol extract of DBD flesh may exert gastroprotective effects through anti-inflammatory and antioxidative mechanisms accompanied with the upregulation of gastroprotective factor PGE_2_ production in the gastric mucosa [38].

PGE_2_ is known to stimulate the secretion of gastric mucus, elevate mucosal blood flow, protect mucosal cells from apoptosis, and accelerate epithelial wound repair and mucosal healing through the activation of prostaglandin E receptors [29,39]. Ethanol administration reduces the gastric mucosal PGE_2_ content [40]. Consistent with the observations reported in the previous studies, our results also demonstrated that PGE_2_ protein content decreased in stomach homogenate by ethanol infusion. However, pretreatment with water or ethanol extracts of either DBD flesh or peel significantly recovered the PGE_2_ level, indicating that the gastroprotective effect of the DBD extract was, at least in part, mediated via PGE_2_.

Multiple studies have identified several compounds obtained from DBD or its derivatives (tuberous root, bark, or fermentates) with pharmacologically potent activities [30]. Considering the previous reports on certain bioactive components found in the whole tuber of DBD, such as glycoproteins in water extracts [41] or 6-hydroxy-2,7-dimethoxy-1,4-phenanthraquinone in methanol extract [42], the anti-inflammatory activities of the DBD extract, are presumed to be associated with these components. However, further study is necessary to identify the substances in the DBD extracts responsible for the gastroprotective effect from ethanol-induced ulcers.

Taken together, our findings demonstrate that oral supplementation with the DBD extract, especially ethanol extract of flesh, prior to acidified ethanol administration, may effectively attenuate inflammatory response and enhance antioxidant activities in gastric mucosa and, thereby, alleviating the severity of gastric ulcer. 

## Figures and Tables

**Table 1:** Experimental groups of ICR mice.

Group No.	Treatment ^1)^			Dose (mg/kg BW)
1	Normal	Vehicle		-
2	Ulcerated ^2)^	Vehicle		-
3		OMEP ^3)^		20
4		Flesh	WE ^4)^	100
5				200
6			EE-60 ^5)^	100
7				200
8			EE-95 ^6)^	100
9				200
10		Peel	WE	100
11				200
12			EE-60	100
13				200
14			EE-95	100
15				200

^1)^ The samples were dissolved in the vehicle (5% (*v*/*v*) Tween-80, 10% (*v*/*v*) polyethylene glycol, 10% (*v*/*v*) DMSO, and 10% ethanol in saline) and orally administered to the mice at the designated doses. ^2)^ Gastric ulceration was induced by intragastric instillation of acidified ethanol (60% ethanol in saline containing 150 mM HCl; 200 μL/mouse). ^3)^ OMEP, omeprazole used as a positive control. ^4)^ WE, water extract; the sample extracted using distilled water. ^5)^ EE-60, ethanol extract; the sample extracted using 60% ethanol. ^6)^ EE-95, ethanol extract; the sample extracted using 95% ethanol. ICR: Institute of Cancer Researc.
